# The Pandemic Stressor Scale: factorial validity and reliability of a measure of stressors during a pandemic

**DOI:** 10.1186/s40359-022-00790-z

**Published:** 2022-04-08

**Authors:** Annett Lotzin, Ronja Ketelsen, Irina Zrnic, Brigitte Lueger-Schuster, Maria Böttche, Ingo Schäfer

**Affiliations:** 1 Department of Psychiatry and Psychotherapy, University Medical Center Hamburg-Eppendorf, Martinistr. 52, 20246 Hamburg, Germany; 2 Department of Psychology, MSH Medical School Hamburg, Hamburg, Germany; 3 Unit of Psychotraumatology, Faculty of Psychology, University of Vienna, Vienna, Austria; 4 Division of Clinical Psychological Intervention, Freie Universität Berlin, Berlin, Germany

**Keywords:** COVID-19, Pandemic, Stressors, Psychometrics

## Abstract

**Background:**

This study aimed to assess the factorial validity and reliability of the Pandemic Stressor Scale (PaSS), a new measure to assess the severity of distress for different stressors relevant during a pandemic or epidemic.

**Methods:**

The PaSS was administered in N = 2760 German participants. Exploratory factor analysis was used to extract factors. The factor structure obtained in the German sample was examined in N = 1021 Austrian participants using confirmatory factor analysis. χ^2^, RMSEA, SRMR, CFI, TLI were assessed as global goodness of fit indices for two models (Model 1: nine-factor model; Model 2: nine-factor model combined with a second-order general factor). We additionally assessed factor loadings, communalities, factor reliability, discriminant validity as local fit indices. Internal consistency, item discrimination, and item difficulty were assessed as additional test quality criteria.

**Results:**

The results of the exploratory factor analysis suggested a nine-factor solution with factor loadings accounting for 50.4% of the total variance (Factor 1 ‘Problems with Childcare’, Factor 2 ‘Work-related Problems’, Factor 3 ‘Restricted Face-to-Face Contact’, Factor 4 ‘Burden of Infection ‘, Factor 5 ‘Crisis Management and Communication’, Factor 6 ‘Difficult Housing Condition’, Factor 7 ‘Fear of Infection’, Factor 8 ‘Restricted Access to Resources’, Factor 9 ‘Restricted Activity’). The confirmatory factor analysis showed a sufficient global fit for both tested models (Model 1: χ^2^ (369, *N* = 1021) = 1443.28, *p* < .001, RMSEA = .053, SRMR = .055, CFI = .919, TLI = .904; Model 2: χ^2^ (396, *N* = 1021) = 1948.51, *p* < .001, RMSEA = .062, SRMR = .074, CFI = .883, TLI = .871). The results of the chi-square difference test indicated a significantly better model-fit of Model 1 compared to Model 2 (∆χ^2^ (27, *N* = 1021) = 505.23, *p* < .001). Local goodness of fit indices were comparable for both tested models. We found good factor reliabilities for all factors and moderate to large factor loadings of the items as indicators. In Model 2, four first-order factors showed small factor loadings on the second-order general factor.

**Conclusion:**

The Pandemic Stressor Scale showed sufficient factorial validity for the nine measured domains of stressors during the current COVID-19 pandemic.

**Supplementary Information:**

The online version contains supplementary material available at 10.1186/s40359-022-00790-z.

## Introduction

The COVID-19 pandemic has affected the global population. Lockdown measures such as working from home, physical distancing, reduced social contact, and mask-wearing were implemented to reduce the spread of COVID-19. These measures are related to multiple stressors, such as organizing home office, combining childcare with working from home, reduced leisure possibilities, and restricted face-to-face contact [1–3]. Furthermore, the preventive measures have adversely impacted the economy [4]; many people face financial difficulties and are strained by financial worries [5]. These multiple stressors have been associated with increased levels of heightened levels of psychological distress [3, 6, 7], depression [3, 8, 9], anxiety [10, 11], and posttraumatic stress disorder [PTSD; 12 during the early phase of the pandemic in the general population.

To better understand the mental health burden of the COVID-19 pandemic and to identify high-risk groups, a validated measure of the pandemic-specific stressors is needed. Such a measure could examine which stressors are most burdensome and how these stressors impact wellbeing and mental health. During the COVID-19 pandemic, several measures have been developed to capture distress or anxiety, sleeping problems, or posttraumatic stress symptoms during the pandemic. Examples include the ‘COVID Stress Scales’ [CSS; 13, the ‘Fear of COVID-19 Scale’ [FCV-19S; 14, the ‘Coronavirus Anxiety Scale’ [CAS; 15, and the ‘COVID-19 Burnout Scale’ [COVID-19 BS; 16. These measures assess the perceived global level of distress during the COVID-19 pandemic, but do not distinguish between the burden of different stressors.

Few measures have been developed to assess the stressfulness of different pandemic-related stressors. The ‘Stressors of COVID-19 Scale’ [17] is a 19-item questionnaire measuring the perceived stressfulness of COVID-19 stressors on 5-point scales. The measure covers four domains, including disease-related stressors (e. g. 'I am worried that I will be infected’); Information-related stressors (e. g. ‘I heard some negative news about COVID-19’); Public health measure-related stressors (e. g. ‘Academic schedule was disrupted’); and Environmental stressors (e. g. ‘I am separated and alienated from my classmates and friends’). The measure was developed in a study of Chinese college students. A first validation study exists for the Chinese version of the questionnaire, with Cronbach’s α of 0.94 for the total score [17]. The questionnaire does not cover the domains of home-related stressors (e.g., restricted housing condition, conflicts at home, lack of childcare) and work-related changes.

Kujawa et al. [18] developed the ‘Pandemic Stress Questionnaire’ (PSQ) to assess pandemic-related stressors and severity of distress on 5-point scales. The 25 items are grouped into six subscales (General life disruption, Interpersonal, Financial, Educational/professional goals, Health-self, and Health-others). In a US sample, good internal consistencies of the total severity score (α = 0.79) and convergent validity with the Perceived Stress Scale [19] were reported, but no further data about the validation of the measure could be identified. The questionnaire does not consider COVID-19 infection-related stressors (e.g., fear of contracting COVID-19), and stressors related to the government’s response (e.g., crisis communication).

The ‘COVID-19 Stressors Scale’ [20] is a 23-item questionnaire that assesses exposure to COVID-19 specific stressors and severity of burden on 5-point scales within the past week on three subscales (‘Infection-related stressors’,’Daily activity-related stressors’, and ‘Financial/resource-related stressors’). The scale was evaluated by using an Exploratory Factor Analysis (EFA) that yielded a one-factor solution accounting for only 21.76% of the variance [21]. Internal consistency (Cronbach’s α = 0.96) was high, while factor loadings were moderate to high (item-total correlations 0.61–0.86), and item discrimination indices were moderate to good (r_it_ = 0.46–0.74). The questionnaire showed convergent validity with the Perceived Stress Scale [19] and Generalized Anxiety Disorder Scale-7 [GAD-7; 22. The measure assesses restricted social contact by only one item, although restricted social contact seems to be a key stressor during the COVID-19 pandemic [23–25].

Another measure assessing exposure to pandemic-specific stressors is the 21-item ‘COVID-19 Stress Scale’ [CSS; 26. The CSS measures the frequency of feelings and thoughts about different stressors on 5-point scales (‘Almost never’ to ‘All the time’). An EFA has been conducted based on the data of a convenience sample in India, which revealed five factors that explained 55.27% of the variance (Vexation with others, Immediate concerns, Routine disruption, Uncertainty about the future, and Systematic stressors [26]. The EFA has not been confirmed with confirmatory factor analysis (CFA) in a different sample. Internal consistency for the total score was high (α = 0.90) and ranged between 0.69 and 0.85 for the subscales. The measure does not capture home-related stressors, e.g., conflicts at home or problems with childcare.

The ‘COVID-19 Stressors Score’ has been developed for a study by Ettman et al. [27] to assess the cumulative exposure to 13 pandemic-specific stressors. To our knowledge, no psychometric evaluation has been conducted. In addition to these measures, several unnamed questionnaires have been used to acquire pandemic-specific stressors [28–30] that have not been psychometrically tested.

For all questionnaires summarized above, limited or no evidence on their factorial validity could be identified. Psychometric information most often concerned internal consistency assessed with Cronbach’s α [22]. Two studies performed an EFA [21, 26], but no CFA has been conducted on any of the identified measures to confirm the dimensional structure. Furthermore, no measure has been psychometrically evaluated in more than one study. Many measures leave out important stressors such as difficult housing conditions. To assess the multiple stressors of a pandemic or epidemic, the psychometric examination of such a measure is needed. Therefore, this study aimed to assess the factorial validity and reliability of the Pandemic Stressor Scale, a newly developed measure of assessing specific stressors during a pandemic or epidemic.

## Methods

### Initial scale development

We developed the Pandemic Stressor Scale (PaSS) to measure the severity of the burden of different stressors relevant during a pandemic or epidemic. After reviewing the previous literature on pandemic-related stressors, a clinical psychologist in trauma and stress researcher (first author) constructed an item set, which was reviewed, reduced and revised in two review rounds by an international consortium of trauma and stress experts that were members of the European Society of Traumatic Stress Studies (ESTSS). The expert group included 22 researchers from 11 countries that belong to the ADJUST study consortium, including researchers with expertise in scale development (for information about the members of the consortium, please see https://doi.org/10.17605/OSF.IO/8XHYG). The first draft of questionnaire was reviewed and commented by all researchers of the consortium. Afterwards, the comments on each of the items were reviewed and discussed, and revisions were decided on consensus. The updated version was then reviewed and discussed again and finally consented. This English language questionnaire was then translated by a native German speaker. A second native German speaker checked the correctness of the German translation and both speakers together consented on a final German version.

The questionnaire contained 43 stressors related to a COVID-19 infection (e.g., fear of contracting COVID-19); governmental response and availability of resources (e.g., poor information from the government); staying at home (e.g., difficulties with combining work with childcare); public-life restrictions (e.g., restricted leisure activity); face-to-face Contact restrictions (e.g., restricted personal contact to loved ones); or work (e.g., financial and job loss).

We used the following instruction to assess the severity of the burden of the 43 stressors: ‘Please indicate how much the following things have burdened you *due to the coronavirus pandemic* within the last month.’ The items were rated on five-point scales ranging from 0 to 4 (0 = ‘Not at all burdened’; 1 = ‘Somewhat burdened’; 2 = ‘Moderately burdened’; 3 = ‘Strongly burdened’, 4 = ‘Does not apply to me’). Higher scores indicate a greater burden.

### Study design

This study is a secondary cross-sectional analysis that used data from a longitudinal cohort study to investigate relationships between stressors and symptoms of psychological adjustment disorder during the COVID-19 pandemic [31]. Data reported in this manuscript were drawn from two study sites of the more extensive study (Germany and Austria). All participants provided informed consent to participate in the study. The study was registered in a study registry before starting the study (https://doi.org/10.17605/OSF.IO/8XHYG).

### Study samples and procedures

Participants were drawn from the general populations of Germany or Austria that (1) were at least 18 years of age and (2) willing to participate in the study. We collected data from June to November 2020. Given the pandemic situation, recruitment was predominantly conducted online. We promoted the study via social platforms (e.g. Facebook, Twitter, Instagram, and WhatsApp), leisure and interest groups (e.g. bicycle or car clubs), newsletters (e.g. newsletters of large companies), and via advertisements in newspapers and magazines. We also disseminated the study information through universities, stakeholders, and professional organizations. As this study is a secondary analysis of data derived from a larger study [31], no a-priori sample size calculation was conducted.

### Measures

The Pandemic Stressor Scale (PaSS) is a self-report questionnaire to assess the severity of the burden for different stressors relevant during a pandemic or epidemic. The items are rated on four-point scales ranging from 0 to 4 (0 = ‘Not at all burdened’; 1 = ‘Somewhat burdened’; 2 = ‘Moderately burdened’; 3 = ‘Strongly burdened’, 4 = ‘Does not apply to me’). Higher scores indicate a greater burden. Completion of the questionnaire takes about 10 min. The initial questionnaire contained 43 items which was reduced to 30 items PaSS, Additional file 1: Suppl. 3). Sociodemographic characteristics were assessed by self-constructed items.

### Data analysis

First, we conducted an EFA to initially investigate the dimensional structure of the PaSS in a German sample. Afterwards, a CFA was conducted to examine whether the factorial structure obtained by EFA could be replicated in another sample.

### Study 1: exploratory factor analysis

To examine the factor structure of the PaSS, we conducted an exploratory factor analysis (EFA). The initial EFA included 43 items measuring different pandemic stressors in a German sample of *n* = 2760 participants. Missing data per variable ranged from 0 (0.00%) to 3 (0.11%). Missing values were imputed by using the Expectation–Maximization algorithm of SPSS 27. Before conducting an EFA, the category 4 = ‘Does not apply to me’ category was recoded to 0 = ‘Not at all burdened’. The EFA was done following current recommendations [32]. Maximum Likelihood was used as the extraction method, as recommended when measures will be used with other datasets in the future [28]. An oblimin rotation with Kaiser normalization was applied. As suggested by Child [29], we removed items with communalities of less than 0.20. Afterwards, the EFA was re-run.

We determined the optimal number of factors using the following criteria: (1) Kaiser’s criterion (eigenvalue > 1); (2) a solution of a maximum number of factors with at least two items with a loading greater than 0.40 and a low cross-loading. Items with factor loadings < 0.40 and/or cross-loadings > 75% were removed, starting with the one with the lowest absolute maximum loading on all the factors. The analyses were conducted in SPSS 22.0.

### Study 2: confirmatory factor analysis

To confirm the factorial structure of the PaSS identified in the first sample, a CFA was conducted in a second sample of *N* = 1021 participants. We imputed missing data using the Expectation Maximization procedure. Data analyses were performed using SPSS 27, and SPSS AMOS 26 Graphics. We tested two models based on the results of the EFA. Model 1 consisted of the indicators that represented the items of the questionnaire and nine first-order factors. Correlations between factors were expected and allowed. Model 2 consisted of the same indicators and first-order factors as Model 1 and was extended by a second-order general factor on which all first-order factors loaded.

The Maximum Likelihood (ML) estimation method was used for the CFA. The skew- (γ_1_) and kurtosis index (γ_2_) were used to test for normal distribution of the indicators as a prerequisite for ML estimations;│γ_1_│ ≥ 3.0 and │γ_2_│ ≥ 10.0 were considered as problematic [30], p. 76–77].

#### Global goodness of fit

Indices and cut-offs to examine global model-fit were selected based on recommendations proposed by Schermelleh-Engel [33]: χ^2^ statistics and normed χ^2^ (χ^2^/df; χ^2^/df ≤ 3 rated as acceptable and χ^2^/df ≤ 2 as good), Root Mean Square Error of Approximation (RMSEA ≤ 0.08 rated as acceptable and RMSEA ≤ 0.05 rated as good), and Standardized Root Mean Residual (SRMR ≤ 0.10 rated as acceptable and ≤ 0.05 rated as good).

The Tucker Lewis Index (TLI) and Comparative Fit Index (CFI) > 0.90 were rated as acceptable, taking the large sample size and model complexity into account [34].

#### Local goodness of fit

Local goodness of fit indices were assessed to examine specific parts of the tested models. On the level of indicator, factor loadings (λ ≥ 0.50 rated as moderate, λ ≥ 0.70 rated as large; [34]) and their statistical significance by Critical Ratio (C.R. ≥ │1.96│) were examined. Factor communalities were also assessed (λ^2^ ≥ 0.50 rated as acceptable; [34]).

On the level of the factor, factor reliabilities (FR > 0.60; [35]) were examined. FR is the proportion of variance shared by indicators that estimate a particular latent factor. Indicators belonging to one factor should share much variance. To test evaluate the discriminant validity of the factors, the Fornell-Larcker criterion was used [36]. The Fornell-Larcker criterion compares the Average Variance Extracted (AVE) with the coefficient of determination (R^2^) between two factors. If AVE > R^2^, the two factors are considered discriminant from each other.

#### Additional test quality criteria

Internal consistency of the factors was estimated by Cronbach's α. The number of indicators per factor ratings was based on the recommendations of Ponterotto and Ruckdeschel [37]. Item difficulty coefficients were calculated by dividing the mean of the items by the maximum item score (assumed as good if 0.20 ≤ *p* ≤ 0.80; [38]). The difficulty index of an item indicates how many of the participants answered in the direction of the characteristic. The item difficulty index ranges from 0 to 100; the higher the value, the easier the question. To assess item discrimination, corrected item-total correlations were computed (*r*_*it*_ ≥ 0.30 rated as acceptable and *r*_*it*_ ≥ 0.50 as good; [39], p. 52).

#### Model comparison

To compare Model 1 and Model 2, a chi-square difference test was conducted (∆χ^2^ = χ^2^_Model 2_ − χ^2^_Model 1_ and ∆df = df_Model 2_ − df_Model 1_; [30], p. 281). A conservative significance level of α = 0.01 was chosen because chi-square tests tend to be over-sensitive in large samples [34].

## Results

### Study 1

#### Sample characteristics

The study included *N* = 2760 adult participants from the general German population (Table 1). Seven out of ten participants were female, three out of ten were male. Participants’ age ranged from 18 to 87 years. The education level was high on average. About one out of a hundred participants had been infected with the coronavirus. Seven out of hundred reported a corona infection of loved ones, and one-third of the participants reported knowing someone personally who has been infected. Two out of ten classified themselves as being at risk for severe or life-threatening symptoms of the coronavirus disease.

**Table 1:** Sample characteristics

	German sample *n* = 2760		Austrian sample *n* = 1021	
Sociodemographic variable	*M*	*SD*	*M*	*SD*
Age	40.79	12.43	45.22	14.48
Gender	*N*	*%*	*N*	*%*
Male	792	28.7	345	33.8
Female	1958	70.9	670	65.6
Other	10	0.36	6	0.59
Community				
Large city	1595	57.8	675	66.1
Small city or town	610	22.1	180	17.6
Suburb near a large city	310	11.2	71	6.95
Rural area	245	8.88	95	9.30
Relationship status^a,b^				
Single	618	27.1	213	25.7
Stable relationship (living together)	1423	62.4	496	59.9
Stable relationship (living separately)	194	8.51	93	11.2
Temporary relationship(s)	46	2.02	26	3.14
Education level				
< 10 years of schooling	8	0.29	17	1.67
≥ 10 years of schooling	348	12.6	150	14.7
Vocational studies	996	36.1	319	31.2
Completed studies	1408	51.0	535	52.4
Working situation				
Employed full-time	1351	48.9	558	54.7
Employed part-time	781	28.3	267	26.2
Vocational training or study	414	15.0	116	11.4
Self-employed	99	3.59	74	7.25
Freelancer	76	2.75	31	3.04
Retired	112	4.06	111	10.9
Seeking work	69	2.50	16	1.57
Other	178	6.45	17	1.67
Corona virus infection other				
Loved ones	182	6.59	68	6.66
Someone else I know personally	920	33.3	395	38.7
No	1694	61.4	582	57.0
Coronavirus infection self				
Yes (recovered)	21	0.76	8	0.78
No	2739	99.2	1013	99.2
Risk for severe or life-threatening symptoms of the coronavirus disease				
Yes	589	21.3	164	16.1
No	2171	78.7	857	83.9
Mental disorder diagnosis				
Yes (currently affected)	222	8.04	73	7.15
Yes (recovered)	412	14.9	150	14.7
No	2126	77.0	798	78.2

^a^*n* = 2281 for the German sample

^b^*n* = 828 for the Austrian sample

#### Exploratory factor analysis

The majority of the items were approximately normally distributed, although some items showed a negative skew (Additional file 1: Suppl. 1). The item set of 43 items used in the initial EFA was further reduced, based on their psychometric properties (see methods section). The communalities of the initial EFA solution were > 0.20 for all items, indicating that all items could be retained. Five items with communalities less than 0.2 were removed (Violent assaults at home; Restricted religious or spiritual activities; Increased workload; Restricted work travel; Working in close contact with people who could be infected). Four items with factor loadings < 0.40 were removed (Working from home (home office); Being rejected by others because of own coronavirus infection; Unable to attend the funeral; Restricted physical activity). Four items with cross-loadings > 75% were removed (Loss of daily structure; Being at home most of the time; Having infected others with the coronavirus; Unable to visit loved ones in a critical situation).

The final item set included 30 items. A nine-factor solution showed the best fit with the data and a sufficient interpretability of the factors. The factor solution was confirmed by visual examination of the scree plot. No additional factor exceeded the threshold of an Eigenvalue of 1.0. Each item had a salient loading of > 0.40 on the respective factor. No item of the rotated factor matrix cross-loaded more than 75% on another factor.

The highest factor loadings of the 30 items ranged between 0.437 and 0.949 (Table 2), suggesting meaningful and significant factor loadings [40]. The factors were named as follows: ‘Restricted Face-to-Face Contact', 'Problems with childcare', ‘Work-related problems’, ‘Fear of infection’, ‘Burden of infection’, ‘Restricted activity’, ‘Crisis management and communication', ‘Restricted access to resources’, and ‘Difficult housing condition’.

**Table 2:** Factor loadings in the German sample (N = 2760)

Item	Item description	Problems with childcare	Work-related problems	Restricted face-to-face contact	Burden of infection	Crisis management and communication	Difficult housing condition	Fear of infection	Restricted access to resources	Restricted activity
Item 1	Loss of childcare	0.949								
Item 2	Difficulties with combining work with childcare	0.927								
Item 3	(Threat of) income loss		0.930							
Item 4	(Threat of) job loss		0.833							
Item 5	Reduced working hours		0.688							
Item 6	Not being able to work		0.621							
Item 7	Insufficient financial support by the government		0.603							
Item 8	Restricted face-to-face contact with loved ones			0.841						
Item 9	Restricted face-to-face contact with others			0.758						− 0.499
Item 10	Social isolation			0.798						− 0.458
Item 11	Restricted physical closeness to loved ones			0.734						− 0.340
Item 12	Infection of loved ones with the coronavirus				0.834					
Item 13	Death of a loved one due to the coronavirus inf				0.819					
Item 14	My own infection with the coronavirus				0.486					
Item 15	Poor information from the government					0.845				
Item 16	Poor crisis management of the government					0.786				
Item 17	Media coverage of the coronavirus pandemic					0.458				
Item 18	No place of retreat	0.464					0.883			
Item 19	Conflicts at home						0.639			
Item 20	Restricted housing conditions						0.604			
Item 21	Fear of getting infected with the coronavirus				0.409			0.770		
Item 22	Fear of infecting others with the coronavirus							0.628		
Item 23	Uncertainty about duration and risks of pandemic							0.606		
Item 24	Fear that loved ones get infected							0.437		
Item 25	Restricted access to regular health care/medication								0.720	
Item 26	Restricted access to goods, e.g., food, water								0.559	
Item 27	Insufficient capacity of the health care system								0.486	
Item 28	Restricted leisure activity			0.463						− 0.841
Item 29	Restricted everyday activity			0.416						− 0.648
Item 30	Restricted private travelling									− 0.559

Factor loadings > .40 are reported

### Study 2

#### Sample characteristics

The study included a sample of *N* = 1,021 adults from the general population of Austria (Table 1). The sociodemographic population characteristics were similar to those of the German sample. About two-thirds were female; one-third were male. Participants’ age ranged between 18 and 80 years. The sample had an overall high education level. About one out of a hundred participants had been infected with the coronavirus, seven out of a hundred reported a corona infection of loved ones, and forty out of a hundred knew someone personally who has been infected.

#### Confirmatory factor analysis

All indicators were approximately normally distributed (skew index │γ_1_│ < 3.0 and kurtosis index │γ_2_│ < 10.0; Additional file 1: Suppl. 1; [15], p. 76–77).

### Model 1

#### Global goodness of fit

The χ^2^ statistics showed a statistically significant difference between the model and the observed data (χ^2^ (369, *N* = 1021) = 1443.28, *p* < 0.001, normed χ^2^ = 3.91, Table 3). Both the RMSEA (RMSA = 0.053, *p* = 0.026, 90% CI = 0.051, 0.056) and the SRMR (0.055) showed an acceptable fit. The CFI (0.919) and the TLI (0.904) indicated an acceptable fit of the model.

**Table 3:** Global fit indices of the confirmatory factor analysis for Model 1 and Model 2 in the Austrian Study Sample (N = 1021 participants)

Global Fit Index	χ^2^	df	*p*	normed χ^2^	RMSA	*p* RMSA	RMSA 90% CI	SRMR	TLI	CFI
Model 1	1443.28	369	< .001	3.91	.053	.026	.051–.056	.055	.904	.919
Model 2	1948.51	396	< .001	4.92	.062	< .001	.059–.065	.074	.871	.883

*df* degrees of freedom; *RMSEA* Root Mean Square Error of Approximation; *CI* Confidence Interval; *SRMR* Standardized Root Mean Residual; *TLI* Tucker–Lewis Index; *CFI* Comparative Fit Index

#### Local goodness of fit

All factor loadings were moderate (λ ≥ 0.50) to large (λ ≥ 0.70; Table 4) and significant (C.R. ≥ │1.96│). 16 indicators showed acceptable communalities λ^2^ ≥ 0.50. All factors showed discriminant validity, except for ‘Restricted Activity’, which was not discriminant from ‘Restricted Face-to-Face Contact’. Factor reliability was considered as good with FR > 0.60 for all factors (Table 5).

**Table 4:** Local goodness of fit indices on the indicator level in the Austrian sample (N = 1021)

Scale	Factor loading		Communality	
	Model 1	Model 2	Model 1	Model 2
Problems with childcare				
Item 1	.91	.94	.83	.89
Item 2	.97	.93	.94	.87
Work-related problems				
Item 3	.90	.91	.82	.82
Item 4	.82	.82	.67	.67
Item 5	.59	.59	.35	.35
Item 6	.69	.69	.48	.48
Item 7	.72	.72	.52	.51
Restricted face-to-face contact				
Item 8	.83	.83	.68	.68
Item 9	.75	.74	.56	.55
Item 10	.79	.79	.63	.63
Item 11	.72	.73	.52	.53
Burden of infection				
Item 12	.87	.88	.75	.77
Item 13	.79	.79	.62	.62
Item 14	.55	.54	.31	.29
Crisis management and communication				
Item 15	.84	.84	.71	.71
Item 16	.82	.82	.68	.67
Item 17	.55	.55	.30	.30
Difficult housing condition				
Item 18	.92	.90	.84	.81
Item 19	.67	.66	.44	.44
Item 20	.66	.67	.43	.45
Fear of infection				
Item 21	.61	.60	.37	.36
Item 22	.66	.65	.44	.43
Item 23	.54	.59	.30	.34
Item 24	.80	.78	.64	.61
Restricted access to resources				
Item 25	.69	.72	.47	.52
Item 26	.52	.54	.27	.29
Item 27	.63	.59	.40	.35
Restricted activity				
Item 28	.82	.80	.67	.63
Item 29	.67	.70	.45	.49
Item 30	.57	.56	.32	.32
Pandemic stressor subscales				
Problems with childcare	–	.24	–	–
Work-related problems	–	.41	–	–
Restricted face-to-face contact	–	.79	–	–
Burden of infection	–	.42	–	–
Crisis management and communication	–	.56	–	–
Difficult housing condition	–	.49	–	–
Fear of infection	–	.59	–	–
Restricted access to resources	–	.60	–	–
Restricted activity	–	.72	–	–

**Table 5:** Local goodness of fit indices on the factor level in the Austrian sample (N = 1021)

Scale	FR^a^	AVE^a^	Coefficient of determination (*R*^*2*^)									Discriminant from (AVE > *R*^*2*^)^b^
			CC	WP	RF	BI	MC	HC	FI	AR	RA	
Problems with childcare (CC)	.94	.88	1	.02	.02	.00	.01	.20	.01	.02	.01	All
Work-related problems (WP)	.86	.57	.02	1	.09	.09	.07	.05	.05	.06	.06	All
Restricted face-to-face contact (RF)	.86	.60	.02	.09	1	.08	.16	.14	.21	.17	.48	All
Burden of infection (BI)	.79	.56	.00	.09	.08	1	.04	.01	.39	.12	.01	All
Crisis management and communication (MC)	.79	.56	.01	.07	.16	.04	1	.10	.07	.24	.17	All
Difficult housing condition (HC)	.80 (.79)	.57	.20	.05	.14	.01	.10	1	.05	.05	.11	All
Fear of infection (FI)	.75	.44 (.43)	.01	.05	.21	.39	.07	.05	1	.18	.07	All
Restricted access to resources (AR)	.65	.38 (.39)	.02	.06	.17	.12	.24	.05	.18	1	.15	All
Restricted activity (RA)	.73	.48	.01	.06	.48	.01	.17	.11	.07	.15	1	All, but SC
Pandemic stressors (PS)	.97	.55	–	–	–	–	–	–	–	–	–	–

*FR* Factor reliability; *AVE* Average Variance Extracted

^a^Numbers in brackets indicate differing results for the first-order factors in Model 2, otherwise results were the same for both models

^b^Fornell-Larcker criterion[36]

#### Additional test quality criteria

Internal consistency was excellent for ‘Problems with Childcare’ (α = 0.94), ‘Work-related Problems’ (α = 0.86) and ‘Restricted Face-to-Face Contact’ (α = 0.85), moderate for ‘Burden of Infection’ (α = 0.77), ‘Crisis Management and Communication’ (α = 0.77) and ‘Difficult Housing Condition’ (α = 0.78), and acceptable for ‘Fear of Infection’ (α = 0.74) and ‘Restricted Activity’ (α = 0.72; Additional file 1: Suppl. 2). The ‘Restricted Access to Resources’ factor showed low internal consistency (α = 0.63). Item difficulty indices showed that twelve of the indicators were difficult (*p* < 0.20). Three factors showed average difficulty coefficients of *p* < 0.20 (‘Problems with Childcare’, ‘Work-related Problems’ and ‘Difficult Housing Condition’). Item discrimination indices were evaluated as good (24 items *r*_*it*_ > 0.50) or acceptable (6 items *r*_*it*_ > 0.30). The average item discrimination indices for the factors were good for eight of the nine factors and acceptable for ‘Restricted Access to Resources’.

### Model 2

#### Global goodness of fit

The χ^2^ statistics showed a statistically significant difference between the theoretical model and the observed data (χ^2^ (396, *N* = 1021) = 1948.51, *p* < 0.001, normed χ^2^ = 4.92; Table 3). The RMSEA (0.062, *p* < 0.001, 90% CI = 0.059, 0.065) and the SRMR (0.074) indicated an acceptable model fit. However, the CFI (0.883) and TLI (0.871) showed an insufficient model fit.

#### Local goodness of fit

Factor loadings of the indicators were all moderate to large (Table 4). Four factor loadings of the first-order factors on the second-order general factor were small: ‘Problems with Childcare’ (λ = 0.24), ‘Work-related Problems’ (λ = 0.41), ‘Burden of Infection’ (λ = 0.42) and ‘Difficult Housing Condition’ (λ = 0.49). Three factors showed moderate factor loadings on ‘Pandemic Stressors’: ‘Crisis Management and Communication’ (λ = 0.56), ‘Fear of infection’ (λ = 0.59) and ‘Restricted Access to Resources’ (λ = 0.60). ‘Restricted Face-to-Face Contact’ (λ = 0.79) and ‘Restricted Activity’ (λ = 0.72) showed a large factor loading on the second-order general factor. All factor loadings were significant (C.R. ≥ │1.96│). Acceptable communalities (λ^2^ ≥ 0.50) were found for 17 indicators. Factor reliabilities were similar to those found in Model 1 and were all considered good (FR > 0.60; Table 5). The second-order general factor showed a high factor reliability of FR = 0.97.

#### Additional test quality criteria

The second-order general factor ‘Pandemic Stressors’ showed a moderate internal consistency (α = 0.88; Additional file 1: Suppl. 2). Average item difficulty for this factor was evaluated as good (*p* = 0.30) but was close to the lower bound of the interval (0.20 ≤ *p* ≤ 0.80), indicating an overall high difficulty. For the internal consistencies and item difficulties of the first-order factors, please see the results section for Model 1. Item discrimination indices concerning the second-order general factor were mostly acceptable.

#### Model comparison

The chi-square difference test comparing Model 1 and Model 2 showed a significant difference of the models in their fit to the data (∆χ^2^ (27, *N* = 1021) = 505.23, *p* < 0.001). These results mean that the less complex model (Model 1) fits better to the observed data than the more complex one (Model 2).

## Discussion

Around the world, the COVID-19 pandemic burdens the lives of people. As the duration of the pandemic increases, so does the importance of measuring the adverse effects of stressors on people’s mental health and wellbeing with valid measures. This research aimed to examine the factorial validity and reliability of a new scale, the Pandemic Stressor Scale (PaSS, Additional file 1: Suppl. 3), that aims to assess the different stressors relevant during a pandemic or epidemic. First, an EFA was conducted in a German sample of participants. Second, the dimensional structure identified in the first analysis was examined using CFA in the second sample of Austrian participants to examine its replicability. Global and local goodness of fit indices and additional test quality criteria were evaluated in two models: A nine-factor model (Model 1) and a nine-factor model with a second-order general factor (Model 2).

A nine-factor solution of the PaSS, including 30 items, showed the best fit with the data and sufficient interpretability of the factors. The factor loadings of the 30 items ranged between 0.409 and 0.949, suggesting meaningful and practically significant factor loadings [40]. The identified factors were ‘Restricted Face-to-Face Contact', 'Problems with Childcare', ‘Work-Related Problems’, ‘Fear of Infection’, ‘Burden of Infection’, ‘Restricted Activity’, ‘Crisis Management and Communication', ‘Restricted Access to Resources’, and ‘Difficult Housing Condition’. While five of the nine factors consistently showed low factor loadings on the other factors, four factors showed factors loadings greater than 0.40 on one additional factor. Three items of the factor ‘Restricted Face-to-Face Contact’ (‘Restricted face-to-face contact with others’, ‘Social isolation’, ‘Restricted physical closeness to loved ones’) showed factor loading greater than 0.40 on the ‘Restricted activity’ factor. The factor ‘Difficult housing conditions’ included one item (‘No place of retreat’) that showed loadings greater than 0.40 on the ‘Problems with Childcare’ factor; one item of the ‘Fear of Infection’ factor (‘Fear of getting infected with the coronavirus’) loaded greater than 0.40 on the ‘Burden of infection’ factor. Finally, two items of the ‘Restricted Face-to-Face Contact’ factor (‘Restricted leisure activity’, ‘Restricted everyday activity’) loaded greater than 0.40 on the ‘Restricted Activity’ factor. High cross-loading may indicate that the indicators measure both constructs. However, all items showed lower cross-loadings than 75%, as recommended [40].

All existing questionnaires on pandemic-related stressors were shorter, including between 13 and 25 [17, 18, 41]. These questionnaires might only partly cover pandemic-related stressor domains, such as home-related stressors (e.g., restricted housing conditions, conflicts at home, lack of childcare), or work-related changes. By assessing nine stressor domains with 30 stressors, the PaSS might allow capturing a broader range of stressor domains.

### Confirmatory factor analysis

#### Global fit

The obtained dimensional structure identified in the EFA could be replicated by Model 1. The SRMR, RMSEA global fit indices showed acceptable fit, as well as the TLI and CFI. The χ^2^ statistics of both models indicated significant differences between the theoretical model and the data; however, this measure tends to be overly sensitive in large samples [34]. The global fit of Model 2 was below the threshold we defined for acceptable fit. The comparison of the two models via χ^2^ difference test showed that Model 1 fitted better than Model 2. Consequently, a second-order global factor on which all first-order factors load seems not to be reflected in the data.

#### Local fit

On the level of the indicators, factor loadings of the items as indicators on the first-order factors were moderate to large. However, in Model 2, four of the nine first-order factors had small factor loadings on the second-order general factor. Only the factors ‘Restricted Face-to-Face Contact’, ‘Crisis Management and Communication’, ‘Fear of Infection’, ‘Restricted Access to Resources’ and ‘Restricted Activity’ showed large or moderate factor loadings on a general second-order factor. This result indicates, in line with the significant result of the chi-square difference test, that the computation of a total score might not be reasonable.

Future studies might test models which include more than one second-order factor. While most of the indicators showed acceptable commonalities, some showed low communalities, indicating that a part of the indicator variance remained unexplained by the respective factor.

On the level of the factors, all factors showed acceptable factor reliabilities, including the second-order general factor. The indicators of each factor shared a sufficient amount of variance within this factor, which indicates that they likely measure a similar construct. All factors except the factor ‘Restricted Access to Resources’ discriminated well from each other.

#### Item difficulty

Twelve items had high difficulties, indicating that the respective stressors measured were not considered a severe burden by many participants. This is reflected in the difficulty index of the second-order general factor which is acceptable but close to the lower bound (*p* = 0.30). The high item difficulties in some items resulted in reduced item variance. Most calculations of a CFA are based on variance–covariance matrices that are affected by reduced item variance, resulting in lower global and local fit indices. The restricted item variance in some of the items might have lowered global fit. However, global fit was acceptable in all assessed indices in Model 1. Items with the lowest difficulties might be removed from the questionnaire to increase item variance.

However, while some stressors were not perceived as stressful, it has to be considered that we assessed the data during the summer and autumn time of the first year of the pandemic. For this time period, the burden was reduced, as the lockdown measures were relaxed in both countries and people had the opportunity to spend time outside. Future studies need to reassess the items and their difficulties to a later timepoint of the pandemic, in which the burden of the stressors could be increased, e.g., during a subsequent lockdown period.

#### Item discrimination and internal consistency

Most item discrimination indices were considered acceptable to good, except for three indicators that did not discriminate well between high and low scores of the second-order general factor in Model 2. These results indicate that the items discriminated well between high and low scores of the first-order factor, they discriminated less well concerning the second-order general factor. Internal consistencies were acceptable for all factors except for ‘Restricted Access to Resources’. In Model 2, the second-order general factor showed a moderate internal consistency (α = 0.88).

#### Model comparison

The chi-square difference test showed a better model-fit of Model 1 compared to Model 2. The only difference between the models is the second-order general factor called ‘Pandemic Stressors’. Four of the first-order factors showed small factor loadings on the global second-order factor and less good item discrimination indices for the global factor than those concerning the first-order factors. These results indicate a less well fit of the nine-factor model that includes a second-order general factor, which is consistent with the results found on the level of the local fit indices.

### Strength and limitations

A strength of this study is the use of sufficiently sized samples and the combined use of EFA and CFA in two different large samples to replicate the results obtained in EFA. A limitation of the study is that we used a non-probability sample that was not representative of the general populations of Germany and Austria concerning gender, income, and education. Future studies need to examine the psychometric properties of the PaSS among representative samples. The focus of this study was to assess the factorial validity of the PaSS; hence, we did not examine the convergent and discriminant validity of the measure. The measure’s convergent and discriminant validity with other stressor measures need to be examined in future studies.

We obtained a general population sample which will include participants with physical or mental disorders as they are part of the general population. Future studies might further examine the factorial validity of the PaSS for such populations, for example in people with premorbid physical or mental health conditions, as individuals’ stress levels might differ by such factors [42]. Similarly, individuals infected with COVID-19 might show higher stress levels which might be analyzed separately. In this study, the rate of COVID-19 infected participants was comparably low in this dataset (0.1% currently affected, 1.7% previously infected with COVID-19 [42]), as the data were collected during the first months of the pandemic. Finally, the psychometric properties of the measure should be examined in the general populations of other countries than Germany and Austria.

## Conclusion

Overall, the findings of both analyses empirically support the factorial validity and reliability of the Pandemic Stressor Scale. Further studies might need to examine additional psychometric aspects of the measure.

## Supplementary Information

**Additional file 1:** Supplements.

## Data Availability

Data supporting the findings of this study is available upon reasonable request from the corresponding author.
