# Supplementary material for: The Pandemic Stressor Scale: factorial validity and reliability of a measure of stressors during a pandemic
Source: BMC Psychol. 2022 Apr 8;10:92. doi: 10.1186/s40359-022-00790-z (PMC8990501; doi:10.1186/s40359-022-00790-z)
Supplement: Supplementary file 1 — Additional file 1: Supplements. [file 40359_2022_790_MOESM1_ESM.docx]

**Supplement 1**

Means, Standard Deviations, Skewness and Kurtosis for the Pandemic Stressors Scale Items

|  |  | German sample  (N = 2760) | | | | Austrian sample  (N = 1021) | | | |
| --- | --- | --- | --- | --- | --- | --- | --- | --- | --- |
| Item |  | *M* | *SD* | Skew | Kurt. | *M* | *SD* | Skew | Kurt. |
| Item 1 | Loss of childcare | 0.51 | 1.03 | 1.73 | 1.31 | 0.41 | 0.93 | 2.09 | 2.79 |
| Item 2 | Difficulties with combining work with childcare | 0.49 | 1.02 | 1.78 | 1.47 | 0.44 | 0.96 | 1.95 | 2.17 |
| Item 3 | (Threat of) income loss | 0.92 | 1.15 | 0.81 | -0.89 | 0.53 | 0.96 | 1.62 | 1.23 |
| Item 4 | (Threat of) job loss | 0.82 | 1.13 | 0.98 | -0.62 | 0.41 | 0.89 | 2.06 | 2.88 |
| Item 5 | Reduced working hours | 0.70 | 1.07 | 1.21 | -0.07 | 0.35 | 0.79 | 2.30 | 4.22 |
| Item 6 | Not being able to work | 0.52 | 1.00 | 1.68 | 1.26 | 0.30 | 0.77 | 2.58 | 5.46 |
| Item 7 | Insufficient financial support by the government | 0.46 | 0.89 | 1.85 | 2.17 | 0.41 | 0.86 | 2.06 | 2.99 |
| Item 8 | Restricted face-to-face contact with loved ones | 1.75 | 0.99 | -0.20 | -1.05 | 1.59 | 1.00 | -0.02 | -1.08 |
| Item 9 | Restricted face-to-face contact with others | 1.33 | 0.97 | 0.26 | -0.89 | 1.15 | 0.98 | 0.46 | -0.79 |
| Item 10 | Social isolation | 1.42 | 1.03 | 0.14 | -1.11 | 1.16 | 1.04 | 0.46 | -0.97 |
| Item 11 | Restricted physical closeness to loved ones | 1.46 | 1.10 | 0.09 | -1.31 | 1.31 | 1.08 | 0.26 | -1.21 |
| Item 12 | Infection of loved ones with the coronavirus | 0.87 | 1.10 | 0.85 | -0.78 | 0.92 | 1.11 | 0.79 | -0.85 |
| Item 13 | Death of a loved one due to the coronavirus infection | 0.73 | 1.13 | 1.15 | -0.32 | 0.78 | 1.14 | 1.07 | -0.49 |
| Item 14 | My own infection with the coronavirus | 0.34 | 0.74 | 2.22 | 4.14 | 0.34 | 0.73 | 2.19 | 3.94 |
| Item 15 | Poor information from the government | 0.85 | 0.95 | 0.83 | -0.37 | 0.99 | 0.98 | 0.60 | -0.75 |
| Item 16 | Poor crisis management of the government | 0.82 | 0.96 | 0.91 | -0.29 | 0.96 | 0.97 | 0.64 | -0.69 |
| Item 17 | Media coverage of the coronavirus pandemic | 1.52 | 0.99 | -0.01 | -1.04 | 1.31 | 1.02 | 0.23 | -1.06 |
| Item 18 | No place of retreat | 0.64 | 0.97 | 1.31 | 0.44 | 0.56 | 0.92 | 1.50 | 0.99 |
| Item 19 | Conflicts at home | 0.59 | 0.88 | 1.37 | 0.85 | 0.48 | 0.82 | 1.67 | 1.86 |
| Item 20 | Restricted housing conditions | 0.56 | 0.89 | 1.49 | 1.11 | 0.54 | 0.90 | 1.58 | 1.32 |
| Item 21 | Fear of getting infected with the coronavirus | 1.26 | 0.92 | 0.32 | -0.72 | 1.15 | 0.89 | 0.39 | -0.61 |
| Item 22 | Fear of infecting others with the coronavirus | 1.31 | 1.09 | 0.20 | -1.26 | 1.23 | 1.09 | 0.30 | -1.24 |
| Item 23 | Uncertainty about duration and risks of the pandemic | 2.02 | 0.85 | -0.49 | -0.49 | 1.79 | 0.92 | -0.27 | -0.81 |
| Item 24 | Fear that loved ones get infected with the coronavirus | 1.86 | 0.97 | -0.37 | -0.92 | 1.70 | 1.03 | -0.24 | -1.08 |
| Item 25 | Restricted access to regular health care or medication | 0.47 | 0.76 | 1.66 | 2.12 | 0.66 | 0.87 | 1.11 | 0.25 |
| Item 26 | Restricted access to goods, e.g., food, water, clothing | 0.53 | 0.79 | 1.42 | 1.27 | 0.32 | 0.66 | 2.16 | 4.25 |
| Item 27 | Insufficient capacity of the health care system | 0.83 | 0.98 | 0.89 | -0.38 | 0.87 | 1.02 | 0.80 | -0.64 |
| Item 28 | Restricted leisure activity | 1.58 | 0.99 | -0.06 | -1.04 | 1.33 | 1.00 | 0.17 | -1.04 |
| Item 29 | Restricted everyday activity | 1.40 | 0.94 | 0.16 | -0.86 | 1.04 | 0.91 | 0.48 | -0.67 |
| Item 30 | Restricted private travelling | 1.63 | 1.07 | -0.14 | -1.23 | 1.59 | 1.11 | -0.11 | -1.34 |

**Supplement 2**

Internal Consistency, Discrimination Index and Difficulty Index in the Austrian Sample (N = 1021)

| Scale | Items (*n*) | *M* | *SD* | Cronbach’s α | Item discrimination (*r_it_*) | | | Item difficulty *(P)* | | |
| --- | --- | --- | --- | --- | --- | --- | --- | --- | --- | --- |
|  |  |  |  |  | *M* | Min | Max | *M* | Min | Max |
| Problems with Childcare | 2 | 0.85 | 1.83 | .94 | .88 | .88 | .88 | .14 | .14 | .15 |
| Work-related Problems | 5 | 2.00 | 3.44 | .86 | .69 | .57 | .81 | .13 | .10 | .18 |
| Restricted Face-to-Face Contact | 4 | 5.21 | 3.42 | .85 | .70 | .66 | .75 | .43 | .38 | .53 |
| Burden of Infection | 3 | 2.04 | 2.51 | .77 | .63 | .49 | .71 | .23 | .11 | .31 |
| Crisis Management and Communication | 3 | 3.27 | 2.45 | .77 | .60 | .47 | .67 | .36 | .32 | .44 |
| Difficult Housing Condition | 3 | 1.57 | 2.20 | .78 | .62 | .56 | .73 | .18 | .16 | .19 |
| Fear of Infection | 4 | 5.87 | 2.97 | .74 | .54 | .46 | .65 | .49 | .38 | .60 |
| Restricted Access to Resources | 3 | 1.86 | 1.96 | .63 | .45 | .40 | .52 | .21 | .11 | .29 |
| Restricted Activity | 3 | 3.97 | 2.42 | .72 | .54 | .48 | .62 | .44 | .35 | .53 |
| Pandemic Stressors | 30 | 26.63 | 13.59 | .88 | .42 | .25 | .59 | .30 | .10 | .60 |

*Note. r_it_* = corrected item-total correlation

**Supplement 3**

**Pandemic Stressor Scale (PaSS)**

Please indicate how much the following things have burdened you due to the coronavirus pandemic within the last month.

|  | Not at all burdened | Somewhat burdened | Moderately burdened | Strongly burdened | Does not apply to me |
| --- | --- | --- | --- | --- | --- |
| 1. Uncertainty about duration and risks of the coronavirus pandemic | 0 | 1 | 2 | 3 | 4 |
| 1. Poor information from the government | 0 | 1 | 2 | 3 | 4 |
| 1. Poor crisis management of the government | 0 | 1 | 2 | 3 | 4 |
| 1. Restricted access to goods, e.g., food, water, clothing | 0 | 1 | 2 | 3 | 4 |
| 1. Restricted access to regular health care or medication | 0 | 1 | 2 | 3 | 4 |
| 1. Insufficient capacity of the health care system for seriously ill people | 0 | 1 | 2 | 3 | 4 |
| 1. Media coverage of the coronavirus pandemic | 0 | 1 | 2 | 3 | 4 |
| 1. Fear of getting infected with the coronavirus | 0 | 1 | 2 | 3 | 4 |
| 1. My own infection with the coronavirus | 0 | 1 | 2 | 3 | 4 |
| 1. Fear of infecting others with the coronavirus | 0 | 1 | 2 | 3 | 4 |
| 1. Fear that loved ones get infected with the coronavirus | 0 | 1 | 2 | 3 | 4 |
| 1. Infection of loved ones with the coronavirus | 0 | 1 | 2 | 3 | 4 |
| 1. Death of a loved one due to the coronavirus infection | 0 | 1 | 2 | 3 | 4 |
| 1. Restricted everyday activity (e.g., shopping) | 0 | 1 | 2 | 3 | 4 |
| 1. Restricted leisure activity (e.g., restaurant visit) | 0 | 1 | 2 | 3 | 4 |
| 1. Restricted private traveling | 0 | 1 | 2 | 3 | 4 |
| 1. Social isolation | 0 | 1 | 2 | 3 | 4 |
| 1. Restricted face-to-face contact with loved ones | 0 | 1 | 2 | 3 | 4 |
| 1. Restricted face-to-face contact with others | 0 | 1 | 2 | 3 | 4 |
| 1. Restricted physical closeness to loved ones | 0 | 1 | 2 | 3 | 4 |
| 1. Restricted housing conditions (little space) | 0 | 1 | 2 | 3 | 4 |
| 1. Loss of childcare | 0 | 1 | 2 | 3 | 4 |
| 1. Difficulties with combining work with childcare | 0 | 1 | 2 | 3 | 4 |
| 1. No place of retreat | 0 | 1 | 2 | 3 | 4 |
| 1. Conflicts at home | 0 | 1 | 2 | 3 | 4 |
| 1. Reduced working hours / fewer work orders | 0 | 1 | 2 | 3 | 4 |
| 1. Not being able to work | 0 | 1 | 2 | 3 | 4 |
| 1. (Threat of) income loss | 0 | 1 | 2 | 3 | 4 |
| 1. (Threat of) job loss | 0 | 1 | 2 | 3 | 4 |
| 1. Insufficient financial support by the government | 0 | 1 | 2 | 3 | 4 |
